# ET-Network: A novel efficient transformer deep learning model for automated Urdu handwritten text recognition

**DOI:** 10.1371/journal.pone.0302590

**Published:** 2024-05-17

**Authors:** Ameer Hamza, Shengbing Ren, Usman Saeed

**Affiliations:** Computer Science and Engineering, Central South University, Changsha, China; University of Kurdistan Hewler, IRAQ

## Abstract

Automatic Urdu handwritten text recognition is a challenging task in the OCR industry. Unlike printed text, Urdu handwriting lacks a uniform font and structure. This lack of uniformity causes data inconsistencies and recognition issues. Different writing styles, cursive scripts, and limited data make Urdu text recognition a complicated task. Major languages, such as English, have experienced advances in automated recognition, whereas low-resource languages, such as Urdu, still lag. Transformer-based models are promising for automated recognition in high- and low-resource languages such as Urdu. This paper presents a transformer-based method called ET-Network that integrates self-attention into EfficientNet for feature extraction and a transformer for language modeling. The use of self-attention layers in EfficientNet helps to extract global and local features that capture long-range dependencies. These features proceeded into a vanilla transformer to generate text, and a prefix beam search is used for the finest outcome. NUST-UHWR, UPTI2.0, and MMU-OCR-21 are three datasets used to train and test the ET Network for a handwritten Urdu script. The ET-Network improved the character error rate by 4% and the word error rate by 1.55%, while establishing a new state-of-the-art character error rate of 5.27% and a word error rate of 19.09% for Urdu handwritten text.

## Introduction

**Fig 1:**
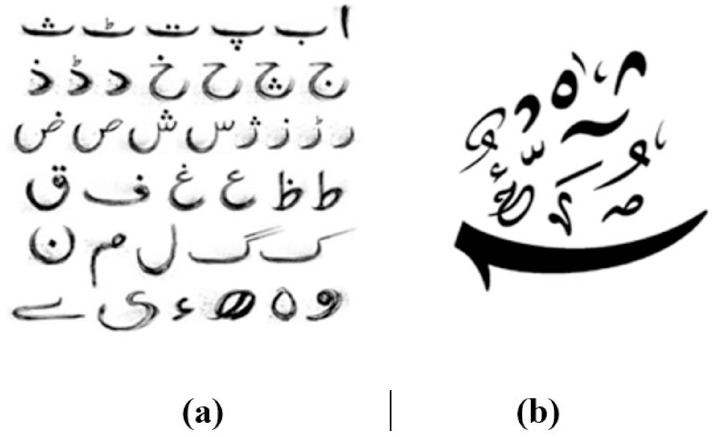
The standard characters of Urdu language (a) Basic Alphabets (b) Secondary characters.

Urdu Handwritten Text Recognition (UHTR) is a technological advancement that focuses on the conversion of handwritten Urdu scripts into machine-readable and editable text. In the modern age of digital technology, computer systems play a key role in the effective storage, processing, and retrieval of data encompassing handwritten documents in the Urdu language. Recognizing handwritten Urdu text is an important task in several applications, including check processing, digitizing historical archives, and understanding a wide range of documents. Urdu is Pakistan’s national language and one of its official languages, with 70.2 million native speakers and 161 million second-language speakers, ranking as the 10th most spoken language worldwide. It is commonly used as a secondary form of communication by many Pakistanis [1]. The Urdu script is complex due to the incorporation of elements from Persian, Arabic, Turkish, Sanskrit, and Portuguese into Urdu requires a delicate balance to maintain linguistic coherence and clarity [2, 3]. Urdu has 45 unique letters and 26,000 ligatures which are more than other languages. This complexity presents significant challenges in Urdu handwritten text recognition due to the absence of word spaces, diagonal writing, contextual variations, and varied styles [4]. Moreover, the large number of unique symbols in Urdu (Fig 1(a)), including diacritics (Fig 1(b)), makes handwritten Urdu text recognition particularly challenging [5, 6]

**Fig 2:**
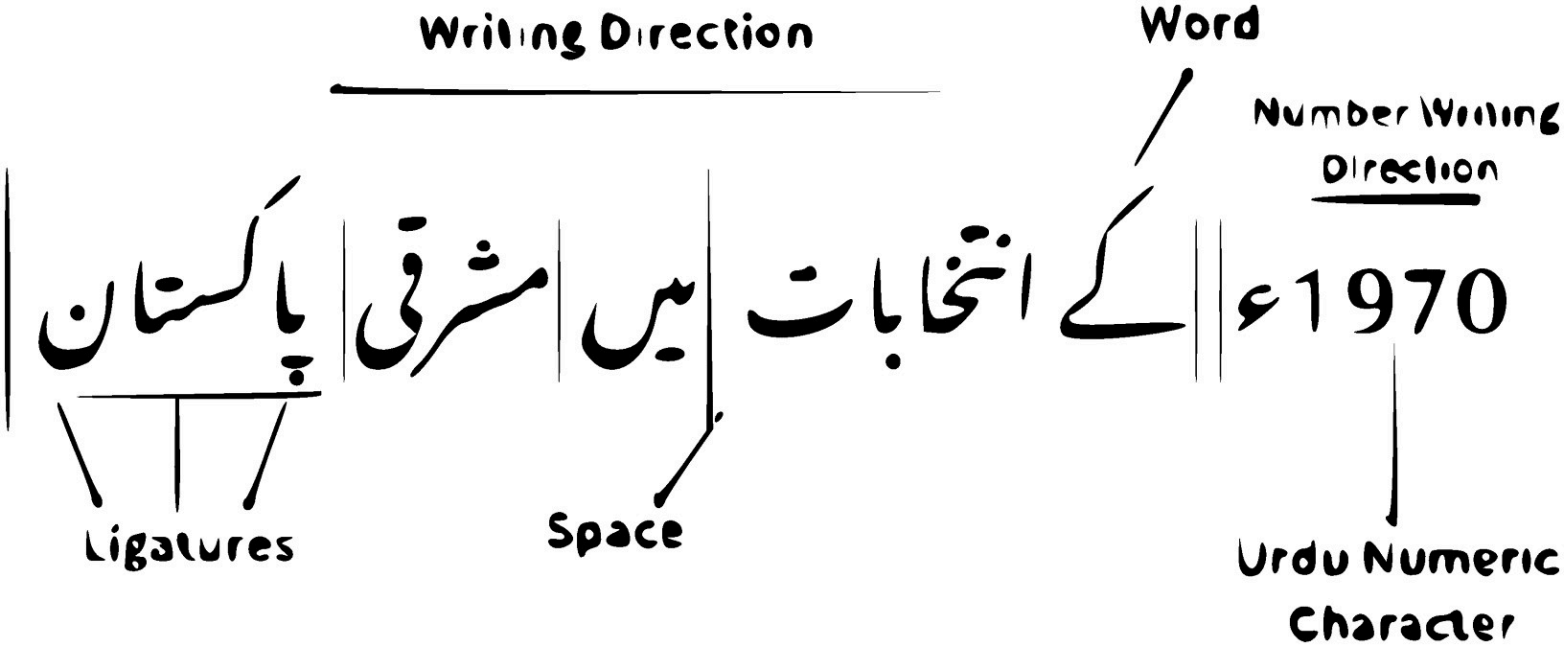
The sample writing style of Urdu language. Numerical characters are left to right, although the writing is right to left.

**Fig 3:**
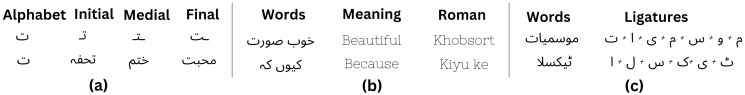
(a) The context sensitivity of Urdu language. (b) Urdu Words Segmentation. (c) Urdu Words ligatures.

Urdu is a cursive language with 12 different writing styles, making text recognition challenging. It is also a bidirectional language, with the script going from right to left for text, and from left to right for numbers, as shown in Fig 2. This makes it difficult for OCR systems to segment Urdu text accurately because spaces are not always used for word boundaries (see Fig 3(b)). Additionally, Urdu characters change their shape depending on their position in a word (Fig 3(a)), making context-sensitive recognition necessary (Fig 3(c)).

Urdu’s handwriting varies significantly from person to person and lacks a standard format, making it challenging to create a universal recognition model. This lack of standardization also leads to inconsistencies in the training data and difficulties in recognition [7, 8]. Deep learning methods such as support vector machines (SVM) [9], convolutional neural networks (CNNs) [10], recurrent neural networks (RNNs), long short-term memory (LSTM) [11], and transformers [12] have made significant progress in improving the accuracy of handwritten Urdu text recognition. However, Urdu text recognition still requires more advanced techniques to address context awareness due to the language’s cursive and bidirectional scripts, handwriting variability, and complicated long-range dependencies [13–15].

This study focuses on offline handwritten Urdu text recognition using a hybrid architecture called ET-Network, which combines EfficientNet with self-attention layers and transformers as a Seq2Seq model. This approach helps to capture local and global features, making it to handle long-range dependencies and ensuring precise identification by preserving the text arrangement. EfficientNet’s [16] scaling techniques are valuable for addressing Urdu’s complex handwriting styles because of the ability to adapt and learn from varying sizes and complexities. Transformers [17] have enhanced the feature representations extracted by EfficientNet with the capabilities of sequential modeling and contextual awareness. The core of language modeling is to take the combined local and global features as input and create contextual representations that lead to the prediction of the next word in a sequence. A prefix beam search is used to evaluate the best possible outcome.

The main contributions of the proposed method are given below:

- To integrate self-attention layers into EfficientNet to capture long-range dependencies in Urdu text.
- To utilize a transformer-based language model that captures context-awareness of Urdu text to improve text recognition.
- To handle handwriting variability in the Urdu language by capturing both local and global context.
- To explore the potential of prefix beam search for improved decoding.

## Related work

Traditional Urdu recognition methods categorize approaches as holistic and analytical [18]. The Roman script employs holistic word-level recognition, whereas Arabic and Urdu use analytical techniques for partial words and characters. Urdu character recognition research often focuses on printed text, with little progress in handwritten recognition. Early printed Urdu script studies were presented by Pal and Sarkar [19]. Their character recognition method employs image processing for feature extraction, segmentation, and recognition. Their system obtained 97.8% character-level accuracy on independent characters.

Urdu’s cursive nature limits separate segmentation, leading to segmentation-free methods like HMM-based approaches [20]. Ud Din et al [21] used statistical features and HMM for ligature recognition. Another method for OCR in Urdu and Arabic without segmentation achieved Google Tesseract-level accuracy [22, 23]. Sagheer et al [24] applied SVM for offline Urdu text recognition with 97% accuracy on the CENPARMI Urdu word dataset, using preprocessing and structural features.

### Deep learning approaches

The methods described in [7, 17, 25] show how convolutional-recursive architectures can effectively recognize recursive text. In the analytical method, Hassan et al. [18] Used a CNN for character segmentation and a Bi-LSTM for classification in handwritten text recognition. Their network had seven convolutional layers, pooling, batch normalization, dropout, and two BiLSTM layers, achieving an average character identification rate of over 83% on 6,000 text lines during testing. The UNHD dataset [26] was utilized for this investigation.

Zia et al. [26] employed CNN, RNN, and interpolated n-gram modeling to recognize handwriting on the ‘NUST-UHWR’ dataset and achieving 5.49% CER. However, LSTM, BERT, and GPT-3 surpass n-gram language models [27, 28]. Therefore, handwriting recognition is a seq2seq task like neural machine translation. In [7], Naz et al. The cutting-edge approach extracted text features using a five-layer CNN. These attributes are fed into a multidimensional long-short LSTM network for context and data sorting. On the UPTI dataset, this method was 98.12% accurate. Husnain et al. [29] CNNs combine structure, geometry, and pixels for Urdu character recognition. Four-layer CNN for feature extraction, fully linked for classification. The authors obtained 96.05% accuracy with 800 Urdu letter and digit pictures. In previous work [11] Offline character recognition using RNN and LSTM models was performed on a dataset of 110,785 handwritten Urdu characters. The accuracy of LSTM was greater than SimpleRNN. The study shows LSTM’s character recognition efficacy, explores character identification problems, and suggests further research. They got 73.19% and 91.80% on simpleRNNN and LSTM respectively. Z.Memon et al. [30] presents a content-controlled GAN-based approach for readable Urdu handwriting. The generator is trained on printed ligatures and fine-tuned using handwritten samples. Discriminators evaluate visual realism, while recognizers assure readability. High-quality Urdu handwriting, improved OCR, and transfer learning are achieved. This paper was 69.70% accurate.

### Transformer based approaches

Attention-based techniques have achieved success in machine translation, image captioning, and speech recognition by enabling the extraction of relevant features from images. In [31], the authors suggested extracting Urdu handwriting using an attention-based method after its success in machine translation and visual description. Identifying character prediction context helps with handwritten text extraction.

Transformers help attention-based models to handle long-range dependencies, enable parallel processing, and increase model flexibility. Shaiq et al. [12] presented a transformer-based Urdu handwritten text recognition model to extract handwritten Urdu text for information preservation. They discussed Urdu script complexity and insufficient resources for Urdu OCR. Their technique uses transformers for interpreting complex handwriting, requiring dataset preprocessing, ResNet18 feature extraction, and transformer-based character prediction. Evaluation with character error rate (CER) suffered by small datasets. The authors recommended testing with pre-trained models and a pre-trained Urdu Language decoder for superior results.

The study [32] introduces a single framework for Urdu printed and handwritten text recognition. A novel CNN block, Transformer encoder, and pre-trained Transformer decoder were used for image analysis and language modeling. The model performed well with varying typefaces and writing styles across datasets. Convolution before the Transformer assisted generalization, and CTC and cross-entropy loss training worked. The model had 6.20% character error rates (CER) on UPTI2.0, URTI, NUST-UHWR, and MMU-OCR-21 and suggested scalability to bigger datasets.

N.Yasin et al. [33] use transformer-based Neural Machine Translation (NMT) to post-process cursive Urdu OCR data with 57% error correction. It discusses Chinese word segmentation, Urdu sentence boundary disambiguation, and neural network and weighted finite state transducer OCR post-processing. It proposes bigger datasets for improvements. AF Ganai and F Khurshid [34] proposed a new Transformer-based BERT architecture-based method for handwritten Urdu text recognition. This article handles cursive script and stroke variance issues. The model fills a gap in unconstrained handwritten Urdu text recognition with excellent word-level identification accuracy and a ligature error rate of 0-10% on multiple datasets.

Researchers in Urdu handwritten text recognition prioritize local features. However, sophisticated features are needed to accurately capture long-range dependencies and contextual information in images. It is needed to capture global features along with local features to solve the limitation of long-range dependencies. The primary objective of the proposed method is to find better computational techniques for feature extraction and recognition in order to address the limitations of current approaches.

## Materials and methods

Inspired by these novel approaches [17, 25, 31], the work addressed Urdu handwritten text recognition as a Seq2Seq modeling challenge. A full transformer model was designed for neural machine translation [17]. This model uses an encoder-decoder with an attention mechanism to develop a language model for the translated text. After obtaining ideas from this, the proposed study applied the Seq2Seq task to handwritten text recognition. The main objective of this study is to take an image as a dynamic sequence, generating digitized text as the output. The transformer adds computational complexity *n*_2_ because of the existence of multihead attention levels, which cause slow processing, especially when dealing with large handwritten text images, where n is the number of sequence lengths.

**Fig 4:**
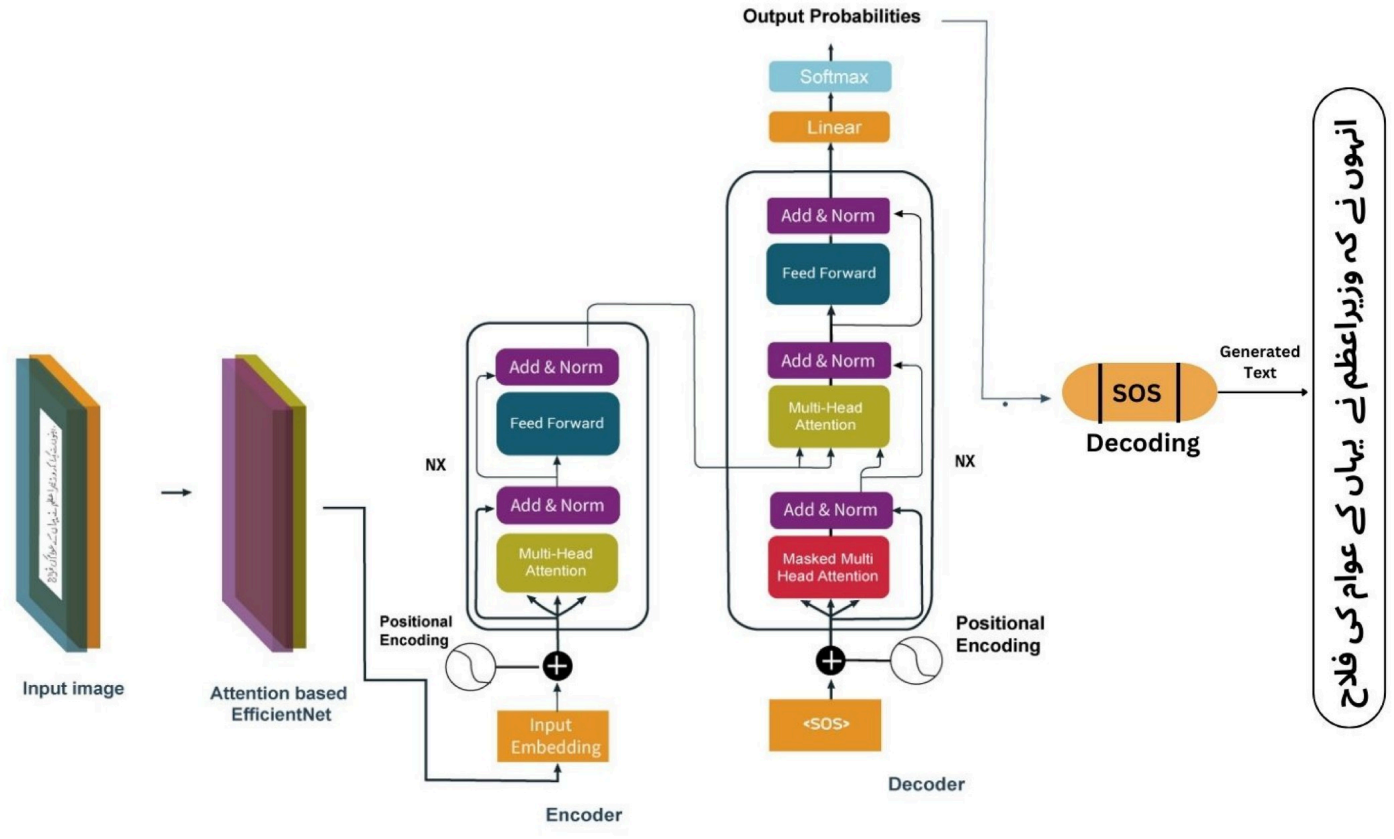
The proposed ET-network architecture for Urdu handwritten text recognition.

**Fig 5:**
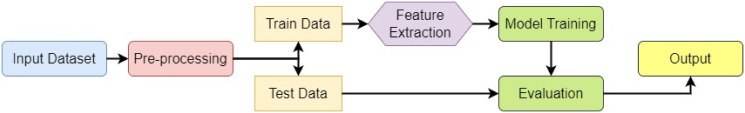
The flowchart for proposed approach for Urdu handwritten text recognition.

To overcome the above-mentioned challenges, a novel Efficient Transformer Network (ET-Network) is proposed for automated recognition of Urdu handwritten text as shown in Fig 4. To extract deep features from the input dataset, a modified version of EfficientNetB0 is used. The standard architecture of EfficientNetB0 consists of 7 blocks in which MBConv blocks are used. However, the proposed version of the EfficientNetB0 contains the first 5 blocks in the fourth block consisting of only two MBConv layers. An attention module is added after each block to focus on the most relevant parts of the input dataset that enhance the accuracy and robustness of the proposed model. These features are passed to the transformer for further processing. The transformer receives the embeddings which are extracted using the modified version of the EfficientNetB0. These embeddings are inserted with the positional information. The locations of the input embeddings are added by using the positional encodings [18]. The transformer turns the suggested model into a language model, eliminating the need for a separate language model, unlike [25]. Three encoder layers followed by three decoder layers are used to make a simple architecture instead of a complicated ones. The tokens from the right-shifted output are fed to the decoder part during the training of the model. The final output, a linear layer followed by the Softmax activation is used to project the decoder embeddings of the model dimension. The prefix beam search decoding is used to predict the best sequence of the output based on the probability of the sequence. The performance of the prefix beam search is more efficient than the greedy decoding. The basic flowchart of the ET-Network is shown in Fig 5.

The ET-Nework architecture is discussed in detail separately below.

### Attention based EfficientNet

This study used the EfficientNet-B0 model from the EfficientNet family as the baseline model because of its good trade-off in terms of dimensions (i.e., number of parameters) and runtime (i.e., FLOPS cost). EfficientNet-B0 consists of many MobileNetV2-like blocks, called MBConv blocks. These blocks contain depth-wise separable convolutions that enable the extraction of features at different scales. Depth-wise separable convolutions decrease the parameters and calculations by splitting the operation into depth-wise and point-wise steps, making them efficient for resource-constrained mobile devices.

**Fig 6:**
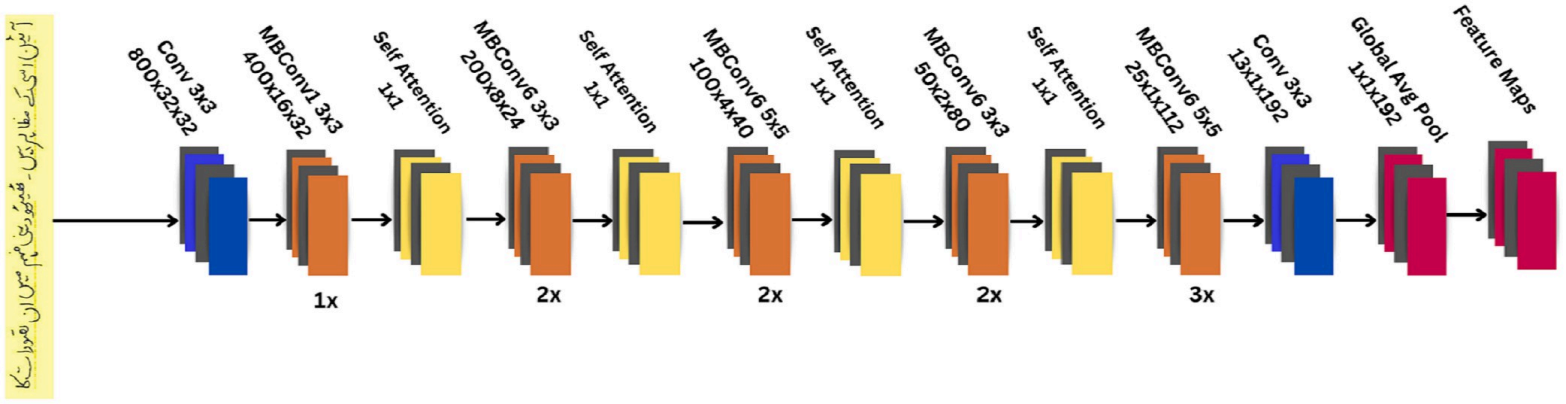
The proposed attention-based EfficientNet-B0 for feature extraction.

The ET-Network utilizes a modified version of EfficientNet-B0 to extract deep features from the input dataset. The standard architecture of EfficientNetB0 consists of 7 blocks in which MBConv blocks are used. However, the proposed version of the EfficientNetB0 contains the first 5 blocks in the fourth block consisting of only two MBConv layers. An attention module is added after each block to focus on the most relevant parts of the input dataset that enhance the accuracy and robustness of the proposed model. The architecture of the modified attention-based efficientnetb0 is shown in Fig 6. This allows the model to capture both fine details in the local regions and long-range dependencies across the image. This is beneficial for recognizing complex patterns in Urdu handwritten characters in large images. The ability of the attention mechanism to learn intricate patterns is responsible for this improvement.

The proposed architecture denotes the input handwritten image as IMG and the extracted features from EfficientNet-B0 as *F*_*eff*_. The attention scores are calculated based on the extracted features *F*_*eff*_. These scores indicate the relevance of each pixel in the image for the recognition process. One way to calculate attention scores is through a convolutional operation, followed by nonlinear activation functions (such as ReLU).

$$\begin{array}{c} Attentionscores=Conv2D(ReLU\left(Conv2D\left(F_{eff}\right)\right)) \end{array}$$ (1)

Conv2D(Feff) performs a 2D convolution operation on the input feature map *F*_*eff*_ and Conv2D(ReLU(Conv2D(Feff))) applies a second convolutional layer to the output of the previous Conv2D layer after passing through the ReLU activation function. This method extracts more complicated data characteristics. Then, the softmax activation function is applied to the attention scores to obtain attention weights that sum to 1. These weights represent the importance of each location in an image for text recognition.

$$\begin{array}{c} Attentionweights=Softmax(Attentionscores) \end{array}$$ (2)

Apply the attention weights to the extracted features to obtain a weighted features map, where each feature is multiplied by its corresponding attention weight.

$$\begin{array}{c} Featuresmaps=F_{eff}\times Attentionweights \end{array}$$ (3)

The weighted features are then summed or averaged along the spatial dimensions to obtain an aggregated feature representation. This procedure enhances the capability of the model to prioritize relevant details within the handwritten image, leading to an overall improvement in text recognition accuracy.

### Transformers

Transformers developed in [17] revolutionized deep learning by adopting an attention mechanism to capture both short- and long-term dependencies. This major development surpasses those of RNNs and LSTMs, which suffer from vanishing gradient difficulties [11]. The transformer design uses self-attention in the encoder and causal attention in the decoder. Tokens in the decoder care only about previous tokens, whereas self-attention allows each input position to look at all others. The multihead encoder-decoder attention significantly accelerated this process. This attention mechanism significantly enhances handwriting tasks by instructing the model on which picture pixels are focused while generating specific characters, as shown in Fig 4.

Query (Q), key (K), and value (V) matrices feed the attention mechanism of the transformer. There are several representations of the input embedding after dense or linear layers. Eq 4 calculates the attention scores as the dot product of the encoder and decoder hidden states (in encoder-decoder attention).

$$\begin{array}{c} Attention\left(Q,K,V\right)=Softmax\left(\frac{QK^{t}}{\sqrt{d_{k}}}\right)V \end{array}$$ (4)

*d*_*k*_ represents the dimensions of key vectors (K). It is a constant used to scale the dot product before applying Softmax to control the magnitude of the attention scores. *K*_*T*_ represents the transposition of key matrix K, which is needed to calculate the dot product of the attention mechanism and attention scores. The square root of the embedding depth scaled the dot product. The softmax approach converts these into probabilities, or focus weights. The V vector multiplies the attention weights to help character producers focus on vital regions. V, K, and Q are partitioned into many attention heads to allow the model to focus on the input from various visual spaces.

In the proposed ET-Network, the encoder component of the transformer gets the embeddings generated from the Attention-based EfficientNet module, which represents the feature maps. Positional information is provided to these embeddings to compensate for the transformer’s encoder’s lack of repetition, which is also lacking in recurrent neural networks. To solve this, positional encoding is implemented as described in [18]. There are three encoder layers placed on top of one another, followed by three decoder levels. The number of layers in the encoder and decoder are determined manually. During the training phase, the right-shifted output tokens are fed into the decoder along with an embedding layer. A linear layer is used to anticipate the final output tokens, followed by a Softmax activation function that projects the decoder’s embedding of model dimensions to match the vocabulary size dimension.

### Prefix beam search

Prefix Beam Search enhances sequence generation in numerous applications by addressing inefficiency, lack of diversity, and inadequate evaluation in classic beam search. It focuses on prefix scoring and ranking, resulting in more efficient, diverse, and high-quality sequence production [35]. Deciphering complex languages with elaborate scripts, such as Urdu, is difficult due to inherent ambiguity in letter and ligature creation. Prefix Beam Search is useful in these situations since it takes into account contextual elements and linguistic limitations to effectively select the most likely character sequence. Prefix beam search, a crucial algorithm for autoregressive models, effectively tackles computational challenges in Urdu handwritten text recognition. By striking a balance between optimality and tractability through the beam width parameter “k,” it strategically narrows down the search space, reducing computational overhead. In the realm of Urdu handwritten text recognition, a tailored version of prefix beam search optimizes calculations by leveraging the algorithm’s pruning aspect, limiting the burden to k * k probabilities. Operating at the character level, this variant capitalizes on the efficiency of a reduced character vocabulary. This character-level approach aligns beam widths more closely with the actual vocabulary size, significantly enhancing accuracy compared to traditional word-level techniques.

$$\begin{array}{c} B_{\left(t+1\right)}=Top_{k}\left(U_{(b\in B_{t}}\right)ExtendedPrefix_{\left(b,i\right)}) \end{array}$$ (5)

Where: *B*_(*t*+1)_: This represents the set of candidate sequences at time step (t+1). *Top*_*k*_: This operation refers to selecting the top-k sequences based on a certain criterion (e.g., highest probability or score). *U*_(*b*∈*B*_*t*_)_: This represents the set of sequences ‘b’ that belong to the set *B*_*t*_, which contains candidate sequences at time step ‘t’. *extended* − *prefix*_(*b*, *i*)_: This notation indicates the extended prefix of a sequence ‘b’ with respect to a certain index ‘i’. The extended prefix is typically the sequence up to the ‘*i* − *th*’ element of ‘b’.

The algorithm’s core equation, expressed embodies the essence of prefix beam search in decoding Urdu sequences. It iteratively explores possible sequences, starting with initial characters after the “BOS” token. The scoring function guides the evaluation and extension of the top k sequences, ensuring the selection of the most probable choices for the next character. The process continues until the “EOS” token is encountered, marking the end of a sequence. To prevent premature sequence termination, a dedicated cache stores the top k finished sequences, safeguarding against their replacement by potentially inferior incomplete strings. This approach ensures that only the most promising complete sequences contribute to the final output, making prefix beam search a powerful and efficient tool for Urdu handwritten text recognition.

**Algorithm 1:** Algorithm of the Proposed ET-Network.

**Require:** Input image I, target sequence T, beam width b

**Input:** Image Dataset

**Output:** Predicted Text

1. Extract features using EfficientNet-B0:

 F ← *extract_features_efficientnet(I)*

2. Apply attention mechanism to image features:

 H ← *apply_attention(F)*

3. Initialize transformer input with positional encodings:

 Transformed *input ← initialize_positional_encodings(H)*

4. Initialize an empty beam of hypotheses Beam:

 for each character c in start token:

  Beam. add((c,), log_probability = 0)

5. while decoding steps < maximum_decoding_steps:

 5.1. Create an empty set of new hypotheses New_Beam:

  for each hypothesis *H*_*i*_ in Beam:

   yt = *H*_*i*_.last_token_generated

   St = *H*_*i*_.log_probability

   if yt is end token:

    NewBeam.add(Hi)

    continue

   5.1.1. Generate next token distribution using transformer:

    P(yt+1—transformedInput, yt)

   5.1.2. Select the top b candidates from the distribution:

    St+1, Yt+1 = select top beam (P(yt+1—transformed input, yt),b)

   for each candidate token yt+1:

    new hi = Hi.extend(Yt+1,St+1)

    new beam.add(new hi)

   5.1.3. Select the top-b hypotheses from new beam:

    beam = select top hypotheses(new beam, b)

6. Select the best hypothesis from beam based on log probability:

    Hfinal = select best hypotheses(Beam)

7. Return the final decoded text:

    decoded text = Hfinal.generated tokens

## Experimental results

In this study, multiple datasets utilized printed and handwritten are included to evaluate the performance of the proposed architecture. Nust-UHWR, UPTI 2.0, and MMU-OCR-21 datasets are used for experimental setup. All three datasets are publicly available.

### Dataset definition

#### NUST-UHWR

The Nust-UHWR dataset contains images with one line of Urdu handwriting and text labels. Individual images with various text styles are shown. UHWR dataset is divided into training, validation, and testing in Table 1. The dataset has 10,000 text lines, not enough to build a strong handwritten text recognition system.

**Table 1:** The statistics of the NUST-UHWR dataset.

Description	Total Numbers
Number of writers	1000
Total number of words	110,940
Number of total samples	10602
Number of training samples	8483
Number of validation samples	1061
Number of testing samples	1061

#### UPTI-2.0

The dataset’s sample images were culled from the web, print media, and books. Including different kinds of Urdu fonts, it includes nearly 120,000 unique text lines in four different scripts. Our primary goal in using this dataset is to train our architecture and show that it can be applied to other datasets.

#### MMU-OCR-21

This dataset was generated as part of the research endeavor to address the volume and diversity challenges for printed Urdu corpora [36]. The dataset is organized into three levels: text line, word, and character. Every line, word, and character is accompanied by an image produced in one of three fonts: Naskh, Nastaleeq, and Tehreer. The corpus is made up of 602,472 jpg image files and 9 CSV files containing the ground truth. MMU-OCR-21 is the largest Urdu printed text dataset. Table 2 describes the UPTI 2.0 and MMU-OCR-21.

**Table 2:** UPTI 2.0 and MMU-OCR-21 data statistics.

Description	Total Numbers
Number of samples in UPTI 20	1,20,000
Number of samples in MMU OCR	602,472

### Preprocessing and data augmentation

**Fig 7:**
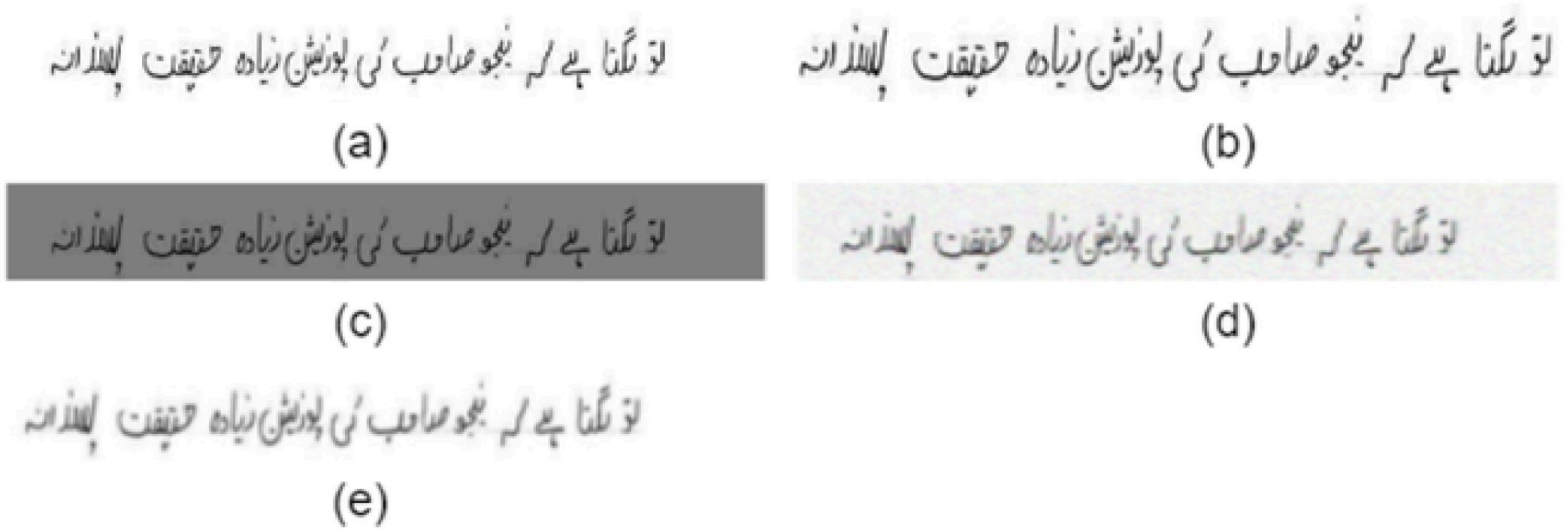
(a) Original image from NUST-UHWR. (b) Cropped image with different dimensions. (c) Brightness adjustment. (d) Adding some soft noise in the image. (e) Slightly blur.

For this study, three separate datasets are combined in which one dataset consists of handwritten texts (NUST-UHWR), and two include printed text (UPTI 2.0, MMU-OCR-21). The merged dataset is divided into three parts: 70% for training, 10% for validation, and 20% for testing. The proposed approach focuses on the handwritten text dataset in test phase. The model was trained on handwritten and printed datasets to extract deep features because of the complicated structure of the Urdu language. The first step before implementation of any deep learning model is to prepare a dataset that forms which is feasible for the model and also causes of saving computation power. The first step is to normalize all dataset images. All images convert into grayscale images and resize with 64px height and 1600 width however maintain the feature ratio. During training, data augmentation is used to address data scarcity and enhance dataset variety. This involves applying transformations like rotation, cropping, and flipping to images. Data augmentation makes the model more robust to input data variations, improving overall performance. In this study, brightness adjustment, cropping, squeezing, added soft noise and applied blur effects were implemented as part of data augmentation. The chosen hyper parameters, including a brightness factor of 1.5, crop percentage of 0.2 for random cropping, squeeze factor of 0.3 for size variations, noise strength of 30 for subtle variations, and blur strength of 5 for Gaussian blurring, aim to create a diverse set of augmented Urdu handwritten images, ensuring robust model training by handling variations in light, spatial resolutions, size, noise, and details The augmented images are shown in Fig 7.

### Evaluation metrics

To evaluate the performance of the proposed model, different evaluation metrics are available. The evaluation metric used in this research is the Character error rate(CER) and Word error rate (WER). Both error rates [37] are metrics used to evaluate the accuracy of speech recognition systems or Optical Character Recognition (OCR) systems. CER measures the percentage of character errors and WER measures the percentage of word error between the recognized text and the ground truth. The equation for calculating CER and WER are:

$$\begin{array}{c} CER=\frac{S+D+I}{N} \end{array}$$ (6)

$$\begin{array}{c} WER=\frac{S+D+I}{N} \end{array}$$ (7)

Where:

S: number of substitutions (the number of characters in the recognized text that are different from the ground truth)

D: number of deletions (the number of characters in the ground truth that are missing in the recognized text)

I: number of insertions (the number of extra characters in the recognized text that are not in the ground truth)

N: total number of characters in the ground truth. The Levenshtein distance method calculates S, D, and I by counting the number of single-character modifications (substitutions, deletions, and insertions) required to change one string of characters into another.

### Experimental setup

To implement the proposed model, ET-Network utilized the PyTorch Python library. ET-Network incorporated a modified version of EfficientNetB0, which employs ReLU activation and features built-in normalization within its MBConv blocks, offering a cost-effective and compatible approach for depthwise convolutions. An attention module is added after each block to focus on the most relevant parts of the input dataset that enhance the accuracy and robustness of the proposed model. The transformer component consisted of 3 encoder and 3 decoder layers, as this configuration demonstrated optimal results within a reasonable computational time frame. Further experimentation with varying encoder and decoder layer counts did not yield performance improvements. Following the transformer, a linear layer reshaped the output to (B × Sq × V), where B denotes batch size, Sq represents output sequence length, and V signifies vocabulary size. Due to dataset size constraints, the vocabulary size encompassed all characters encountered within the training data, complemented by special tokens—PAD (padding), BOS (Beginning Of Sentence), EOS (End Of Sentence), and UNK (Unknown character)—to facilitate character-level handwriting recognition. For both training and validation, we employed Softmax activation coupled with cross-entropy loss. The model’s training was executed on a single Nvidia RTX 3090 GPU, utilizing a batch size of 32. To maintain consistency, we padded output sequences with the pad token to match the length of the longest sequence within each batch. The Adam optimizer was employed for model training, with a learning rate of 0.0003, betas (0.9, 0.98), and epsilon 1e − 9. It takes about 1.5 hours to train the proposed model on the training dataset. Alternative learning rates proved detrimental to training, either leading to loss divergence (higher rates) or sluggish convergence (lower rates). The proposed ET-Network focuses on the Urdu handwritten text recognition therefore, the model is randomly tested on a handwritten text from the Nust-UHWR dataset by using evaluation metrics Character Error Rate (CER) and Word Error Rate (WER).

## Results and discussion

**Fig 8:**
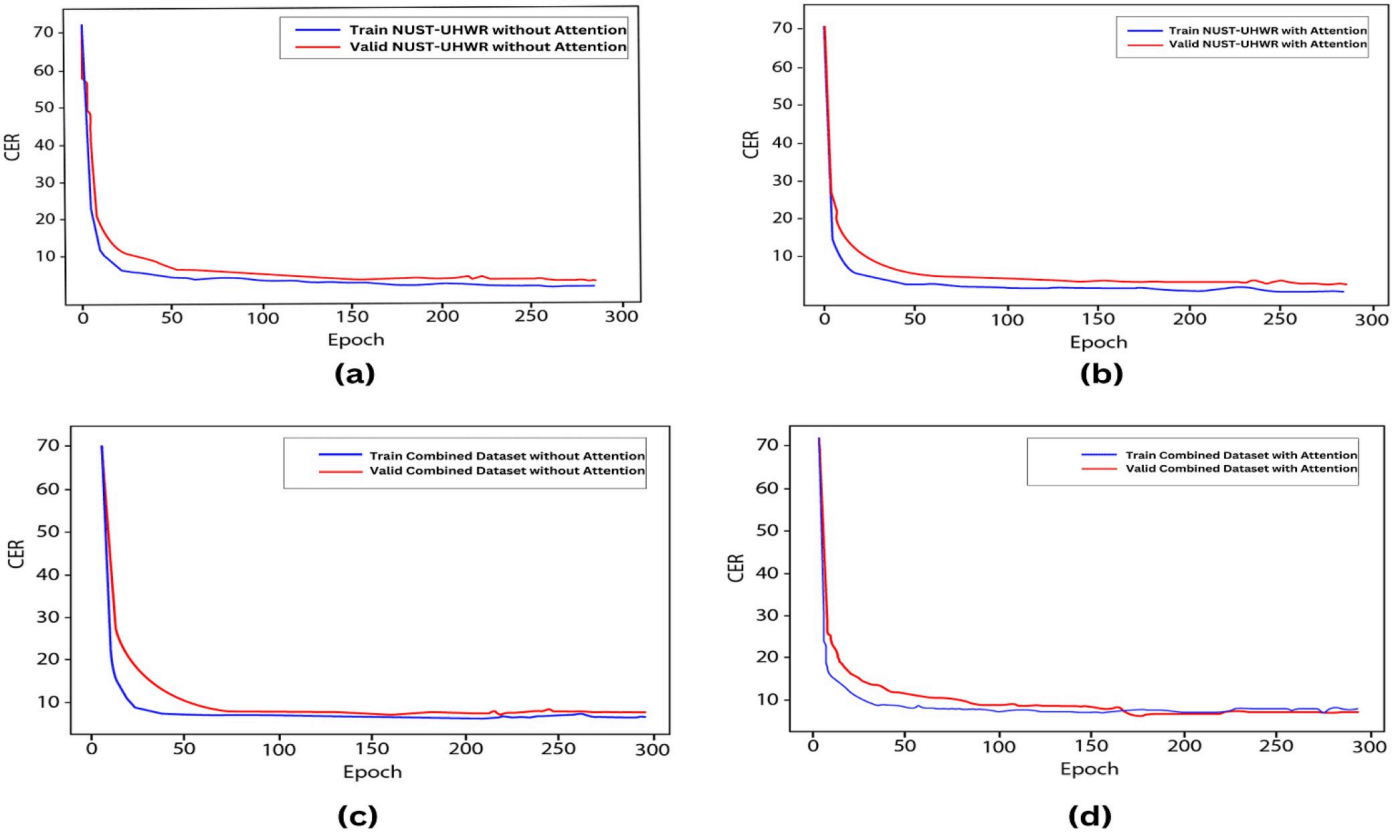
CER training and validation for the proposed architecture. All models is trained at a 0.003 learning rate.

**Fig 9:**
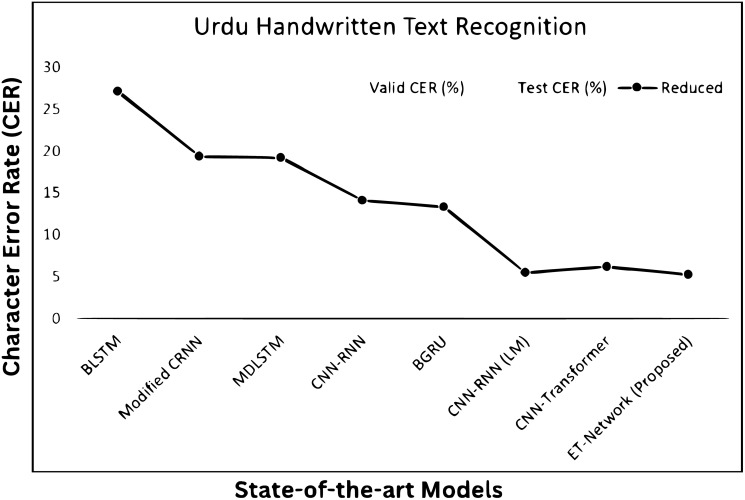
The comparison graph between state-of-the-art methods.

**Fig 10:**
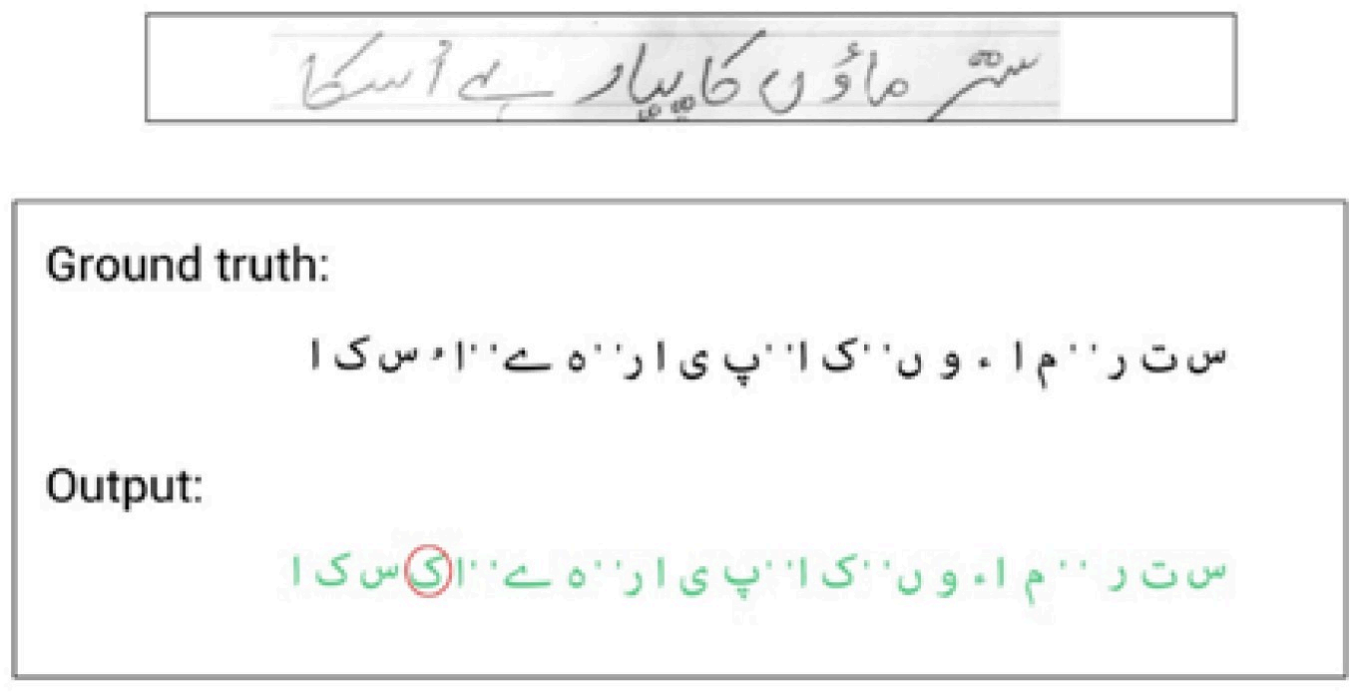
The sample output on random image.

**Fig 11:**
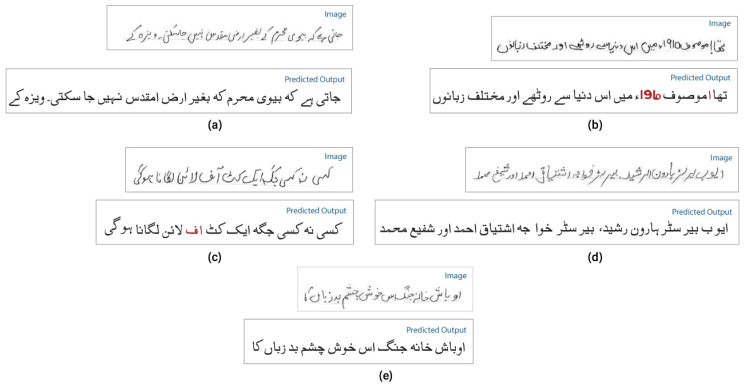
The failure case of proposed ET-Network. (b) The model just incorrectly predicts the number. (c) Model miss the diacritics of the Urdu character.

This part describes a set of tests that are going to be used to verify that the suggested method works. The results are meant to answer the following research questions (RQs):

1. RQ1: Does the EfficientNet with self-attention mechanism effectively capture long-range dependencies?
  The integration of self-attention into EfficientNet-B0 in Urdu handwritten text recognition allows for greater feature extraction flexibility. It enables the model to understand complicated structures and changes within the script, capturing both local and global features in the input image. EfficientNet with self-attention frequently adopts a hierarchical structure with several levels of self-attention layers. This method allows the model to incorporate long-range dependencies at several spatial scales, from local to global, improving its capacity to identify contextual relationships in data. Lower levels are concerned with neighboring pixel connections, whereas higher levels can successfully model dependencies between distant pixels or even contrasted parts of the image. The impact of the Self-attention mechanism in EfficientNet is described in Table 3 with different experiments.
2. RQ2: How does the self-attention in EfficientNet impact the character error rate in Urdu handwritten text recognition?
  Integrating self-attention techniques into EfficientNet for Urdu handwritten text recognition significantly reduces the character error rate (CER) through proper hyperparameter tuning. This enhancement enables the model to capture intricate spatial relationships and dependencies within retrieved image features, particularly valuable for handling the complexities of handwritten Urdu. Improved feature representation boosts recognition accuracy and potentially lowers CER while considering factors like global context, variability, and overfitting reduction, albeit with added computational complexity. Table 4 shows the clear impact on CER (%).
Table 4 presents results with a different number of experiments. It has been observed that adding self-attention layers in EfficientNet to extract better features truly impacts on ET-Network to achieve the best CER. However, the model was slightly overfit with alone NUST-UHWR dataset. The error rate is 5.27% with a self-attention layer after every MBConv block. Different variants of EfficientNet models were tried for some other experiments, but due to the limitation of data, it achieved nothing. The model was more complex and needed more data and time to train. Fig 8 shows CER training and validation for the ET-networks experiments
3. RQ3: Performance of proposed model compare to state-of-the-art methods for Urdu handwritten text recognition?
  The proposed architecture addresses the limitation described above and achieves state-of-the-art results as shown in Fig 9. Table 5 shows the comparative results and Table 6 shows the advantages and disadvantages of existing methods used for Urdu handwritten text recognition.
  A sample image with mistakes is shown in Fig 10. The false predicted character is encircled with red color with a solid outline. The rest of the characters are predicted accurately.
4. RQ4: What are the limitation and failure cases of the model?
  Limitation: Incorporating self-attention layers, such as in EfficientNet, offers advantages in capturing long-range dependencies and reducing CER, however, there are chances for further model enhancements. Firstly, a large and diversified Urdu handwriting dataset is required. When there is plenty of training data, self-attention layers can improve model performance. However, in the lack of appropriate data, their benefits may be limited. Secondly, self-attention layers are computationally expensive, and integrating them with an existing complicated architecture like EfficientNet might require more computer resources for training and inference. Third, handwritten writing is inherently noisy, and while self-attention improves in context, it may also introduce noise into the data. Strong pre-processing and data-cleaning procedures are required. Last but not least, to ensure that the additional self-attention layers fit EfficientNet well without adding unnecessary complexity, careful hyperparameter tuning and architecture design are required otherwise model can be overfit.
  Failure cases: Fig 11 shows some example outputs of the model and failure cases. The model faces difficulty in predicting the numbers because of the writing direction. Fig 11(c) shows how the model predicts wrongly the diacritics. Fig 11(b) shows model predicts wrong numbers and special characters. In other images, the model behaves nicely.

**Table 3:** Ablation study of self-attention layer (SAL). Table evaluate the proposed model with different layers of attention.

Number of Layers	Train CER (%)	Valid CER (%)	Test CER (%)
SAL- 4	5.05	5.14	5.27
SAL- 3	5.21	5.39	5.59
SAL- 2	5.55	5.91	6.03
SAL- 1	5.47	5.87	6.10

**Table 4:** The comparison of CER on valid and test splits with proposed models.

Dataset	Proposed Model	Valid CER (%)	Test CER (%)
Nust-UHWR	ET-Network(No Attention)	7.21	7.79
Nust-UHWR+MMU+UPTI 2.0	ET-Network(No attention)	6.61	6.85
Nust-UHWR	ET-Network(with attention)	5.11	5.70
Nust-UHWR+MMU+UPTI 2.0	ET-Network(with attention)	5.14	5.27

**Table 5:** The comparison of the proposed model with state-of-the-art methods.

Models	Valid CER	Test CER	Test WER
BLSTM [38]	27.39	27.05	–
Modified CRNN [39]	18.57	19.34	–
MDLSTM [40]	14.11	19.15	–
CNN-RNN [41]	13.25	14.12	–
BGRU [42]	13.50	13.28	–
CNN-RNN (LM) [25]	5.28	5.49	19.39
CNN+Transformer [32]	5.90	6.2	–
ET-Network (Proposed)	5.14	5.27	19.09

**Table 6:** Advantages and disadvantages of state-of-the-art methods.

Models	Advantages	Disadvantages
BLSTM [38]	Context-aware processing, Overcomes limitations of traditional RNNs, Segmentation-free approach	Recognition error rate, Dataset variations, Ligature recognition complexity.
Modified CRNN [39]	End-to-end trainability, Unconstrained recognition, Compact model size, Generalizable framework, Competitive performance	Limited applicability, Model complexity, Training data requirements.
MDLSTM [40]	Alphabet-Independent, Globally Trainable, Long-Range Context Access. Hierarchical Structure	Complexity, Training Time, Dimensionality
CNN-RNN [41]	Combined CNN-RNN architecture, Efficiency with CTC, Competitive performance, Contextual information access	Training data requirement, Label size reduction trade-off, Dependence on training data quality
BGRU [42]	Achieves Optimal Character Error Rates, Reduces Overfitting, and Improves Generalization, Potential for Broader Application	Challenges with Certain Handwriting Styles, Scalability Concerns
CNN-RNN (LM) [25]	Top-tier performance, Tackling challenges, Transferable skills	Missing information, Deeper dive into failures
CNN+Transformer [32]	Top-notch performance, Adaptability, Versatility, Robustness	Training data requirement, Label size reduction trade-off, Dependence on training data quality
ET-Network (Proposed)	Improved Feature Extraction, Long-Range Dependency Capture, Hierarchical Structure, State-of-the-Art Results	Noise introduction, Model overfitting, Data limitation

## Ablation study

In this work, an ablation study was conducted to explore the potential of language modeling with different kinds of experiments to obtain the best character error rate (CER). The integration of self-attention into EfficientNet was also explored for better feature representation.

### Ablation study: ET-Network without attention

To explore the potential of the proposed architecture, self-attention layers were removed from the EfficientNet in feature extraction. This study finds that ET-Network model without self-attention is simpler, faster, and more computationally efficient. However, it struggled to capture long-range dependencies, and contextual information and adapt specifically to the Urdu language character from the input image. This scenario directly impacts accuracy in complex cases. ET-Network achieved a 6.85% character error rate (CER) without using self-attention layers. It has been also noticed that if self-attention layers are added into EfficientNet, the CER reduces to 5.27% as shown in Table 7.

**Table 7:** Details of hyper-parameters for proposed ET-Network.

Dataset Split	70% training + 10% validation + 20% testing
Batch size	16
Epochs	100
Learning rate	0.0003 (beta2 = 0.999, epsilon = 0.1)
Dropout	0.2
Optimizer	Adam
Loss function	cross-entropy loss
Activation function	ReLU
Encoder	3
Decoder	3
Embedding dimension	192
Number of Attention heads	8
Prefix Beam width	5
GPU	Nvidia RTX 3090
Input image	1600 16
Training time	1.5 Hours

### Ablation study: Vision transformer(ViT)

**Fig 12:**
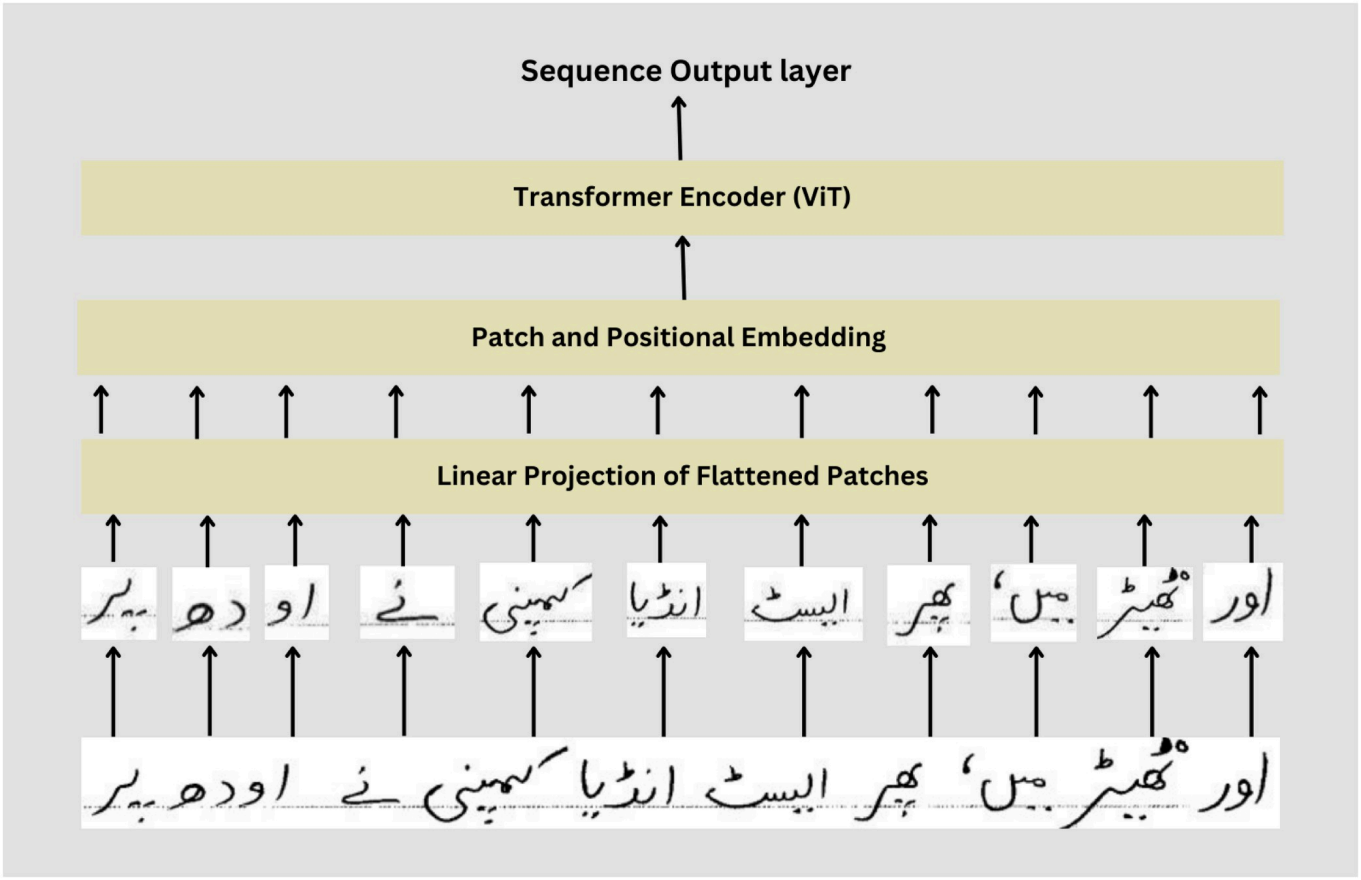
The vision transformer on Urdu text.

In the proposed architecture, we removed EfficientNet to extract features and used a vision transformer [43] for text recognition. Given Urdu text images are divided into non-overlap patches and each patch is treated as a token as shown in Fig 12. Flatten these patches into vectors and add positional embedding to convey spatial information of patches in Urdu text images. These embedded patches feed into a pre-trained vision transformer. We replaced the multi-head classification with a Sequence output layer followed by the softmax activation function. During training, the CTC loss function is used and prefix beam search is used for decoding CTC results into text. Due to data limitations, this architecture has not achieved impressive results, which is why CNN plays a major role in feature extraction. Vision Transformer did not perform well on Urdu text recognition because ViT cannot capture hierarchical features effectively and requires a more diverse dataset however ViT achieves 9-10% CER. ViT models are made for image classification which does not capture contextual information as compared to other Seq2Seq models and ViT is more computationally expensive and memory intensive. Moreover, Vision Transformer struggle with long-range dependencies and domain adaption as well.

## Conclusion

In this paper, the ET-Network proposed for Urdu handwritten text recognizes that utilizes the strength of two deep learning models. EfficientNet with self-attention layers is used to extract local and global features which cause to handle long-range dependencies and contextual information and transform for language modeling. Proposed model test with different experimental setups and datasets and achieved 6.85% Character Error Rate (CER) without using Attention layers. But when integrating attention layers in EfficientNet CER reduced 4% as compared to state-of-the-art methods and got 5.27% CER. Et-Network improves 1.55% word error rate(WER) and achieved 19.09% WER. To the best of our knowledge, this work is a pioneer in using such models for both printed and handwritten recognition. In the future, integrate this architecture with other modalities like audio or images, if available, to enhance recognition accuracy and robustness. The ET-Network can explore innovative attention mechanisms designed specifically for handwritten text recognition tasks, such as hierarchical attention or mechanisms addressing challenges like character segmentation and handling ligatures in Urdu script.
